# Synthesis, crystal structure determination, Hirshfeld surface and crystal void analyses, inter­action energy calculations and energy frameworks of (3a*RS*,4*RS*,9a*RS*)-2-benzyl-3-oxo-2,3,3a,4,9,9a-hexa­hydro-1*H*-benzo[*f*]iso­indole-4-carb­oxy­lic acid

**DOI:** 10.1107/S2056989026005189

**Published:** 2026-05-22

**Authors:** Kseniia A. Alekseeva, Tuncer Hökelek, Victor N. Khrustalev, Maxim Y. Kolomeytsev, Anastasia A. Pronina, Alebel N. Belay, Khudayar I. Hasanov

**Affiliations:** a RUDN University, 6 Miklukho-Maklaya St., Moscow 117198, Russian Federation; b Hacettepe University, Department of Physics, 06800 Beytepe-Ankara, Türkiye; c N. D. Zelinsky Institute of Organic Chemistry, Russian Academy of Sciences, Leninky Prosp. 47, Moscow 119334, Russian Federation; d Department of Chemistry, Bahir Dar University, PO Box 79, Bahir Dar, Ethiopia; e Azerbaijan Medical University, Scientific Research Centre (SRC), A. Kasumzade St. 14, AZ 1022, Baku, Azerbaijan; Universität Greifswald, Germany

**Keywords:** iso­indole, benzo[*f*]iso­indole, IMDAV reaction, Diels–Alder reaction, crystal structure

## Abstract

The title compound, C_20_H_19_NO_3_, consists of a benzyl moiety bonded to the nitro­gen atom of a 1*H*-benzo[*f*]iso­indole-4-carb­oxy­lic acid group. The fused pyrrole and cyclo­hexene rings of the iso­indole group are in envelope and flattened-boat conformations, respectively. The planar benzene rings are oriented at a dihedral angle of 69.64 (3)°. In the crystal, O—H⋯O and C—H⋯O hydrogen bonds link the mol­ecules, enclosing *R*^2^_2_(14) and *R*^2^_2_(9) ring motifs, into infinite double-chains along the *a*-axis direction. π–π stacking inter­actions and C—H⋯π(ring) inter­actions help to consolidate the packing. Hirshfeld surface analysis revealed that the most important contributions for the crystal packing are from H⋯H (52.2%), H⋯C/C⋯H (24.0%) and H⋯O/O⋯H (21.2%) inter­actions. The volume of the crystal voids and the percentage of free space were calculated to be 92.25 Å^3^ and 11.52%, showing that there is no large cavity in the crystal packing. Computational methods revealed O—H⋯O and C—H⋯O hydrogen-bonding energies of −103.8, −82.2 and −35.5 kJ mol^−1^. Evaluations of the electrostatic, dispersion and total energy frameworks indicate that the stabilization is dominated by the electrostatic energy contributions.

## Abstract 2

The asymmetric unit of the title compound consists of a benzyl moiety bonded to the nitro­gen atom of a 1*H*-benzo[*f*]iso­indole-4-carb­oxy­lic acid group. In the crystal, O—H⋯O and C—H⋯O hydrogen bonds link the mol­ecules into infinite double-chains along the *a*-axis direction. π–π stacking inter­actions and C—H⋯π(ring) inter­actions help to consolidate the packing.

## 1. Chemical context

One of the earliest reports on the synthesis of benzo[*f*]iso­indoles *via* a [4 + 2] cyclo­addition was published by Michael T. Cox (1975 ▸). Since then, the intra­molecular Diels–Alder (IMDA) reaction has become a powerful and widely employed strategy for the construction of complex carbo- and heterocyclic scaffolds (Krishna *et al.*, 2022 ▸). IMDA-based approaches enable concise synthetic routes, often providing target compounds in high yields and with significant functional and biological relevance.

The majority of reported IMDA transformations rely on structurally elaborate substrates (Cox, 1975 ▸; Ozawa *et al.*, 2011 ▸; Kim *et al.*, 2014 ▸), in which the dienophilic moiety is typically introduced *via* acyl halide derivatives (Dawson & Mellor, 1995 ▸; Rodríguez *et al.*, 2004 ▸; Bober *et al.*, 2017 ▸). Cyclic anhydrides also exhibit high reactivity in such processes, while the resulting carb­oxy­lic acid functionality offers opportunities for further downstream functionalization (Kolesnik *et al.*, 2025 ▸; Sadikhova *et al.*, 2024 ▸) as well as applications in coordination and supra­molecular chemistry (Huseynov *et al.*, 2021 ▸; Naghiyev *et al.*, 2023 ▸; Mamedov *et al.*, 2006 ▸).

In our previous study, we reported an efficient approach to *N*-alkyl-substituted benzo[*f*]iso­indoles based on the reaction of allyl­amines with maleic anhydride (Alekseeva *et al.*, 2026 ▸). Herein, we present an additional example of this transformation.

**Figure 1:**
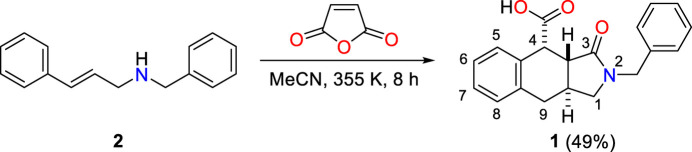
The reaction scheme for the synthesis of the title compound.

The reaction of cinnamyl­amine (**2**) with an equimolar amount of maleic acid anhydride in boiling aceto­nitrile affords the target benzo[*f*]iso­indole-4-carb­oxy­lic acid in satisfactory yield (Fig. 1 ▸). The product crystallizes directly from the reaction mixture and requires no further purification.

It should be noted that establishing the structure and describing the structural features of such a type of compounds is an important task, as a number of derivatives of benzo[*f*]iso­indole are known to be used as bichromophores (Denissen *et al.*, 2017 ▸), plant protections (Song *et al.*, 2023 ▸) and demonstrate the potential of transformation into BODIPY scaffolds (Dvoracek *et al.*, 2025 ▸). Herein, we studied the title compound’s mol­ecular and crystal structures together with its Hirshfeld surface (HS) and carried out crystal void analyses, inter­action energy calculations and energy framework determinations.
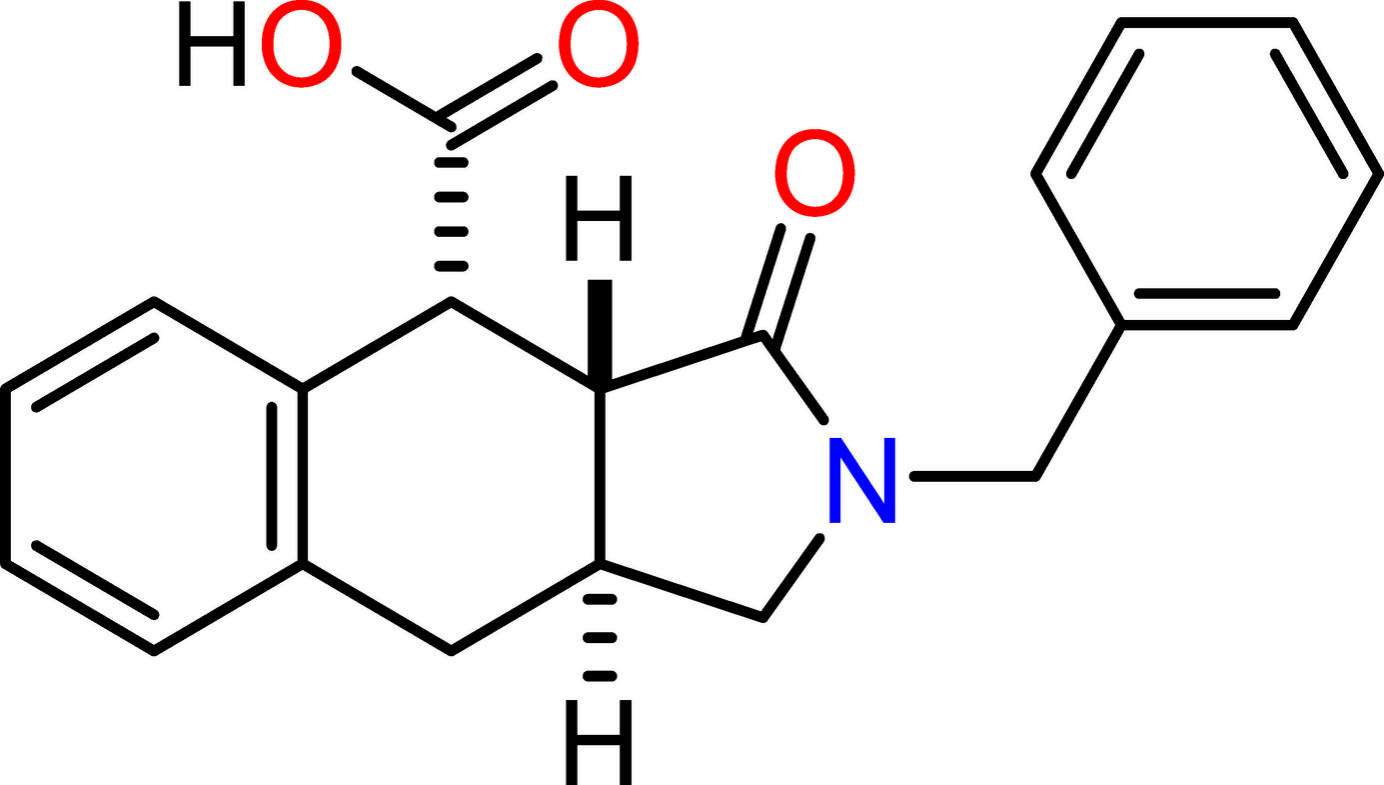


## 2. Structural commentary

**Figure 2:**
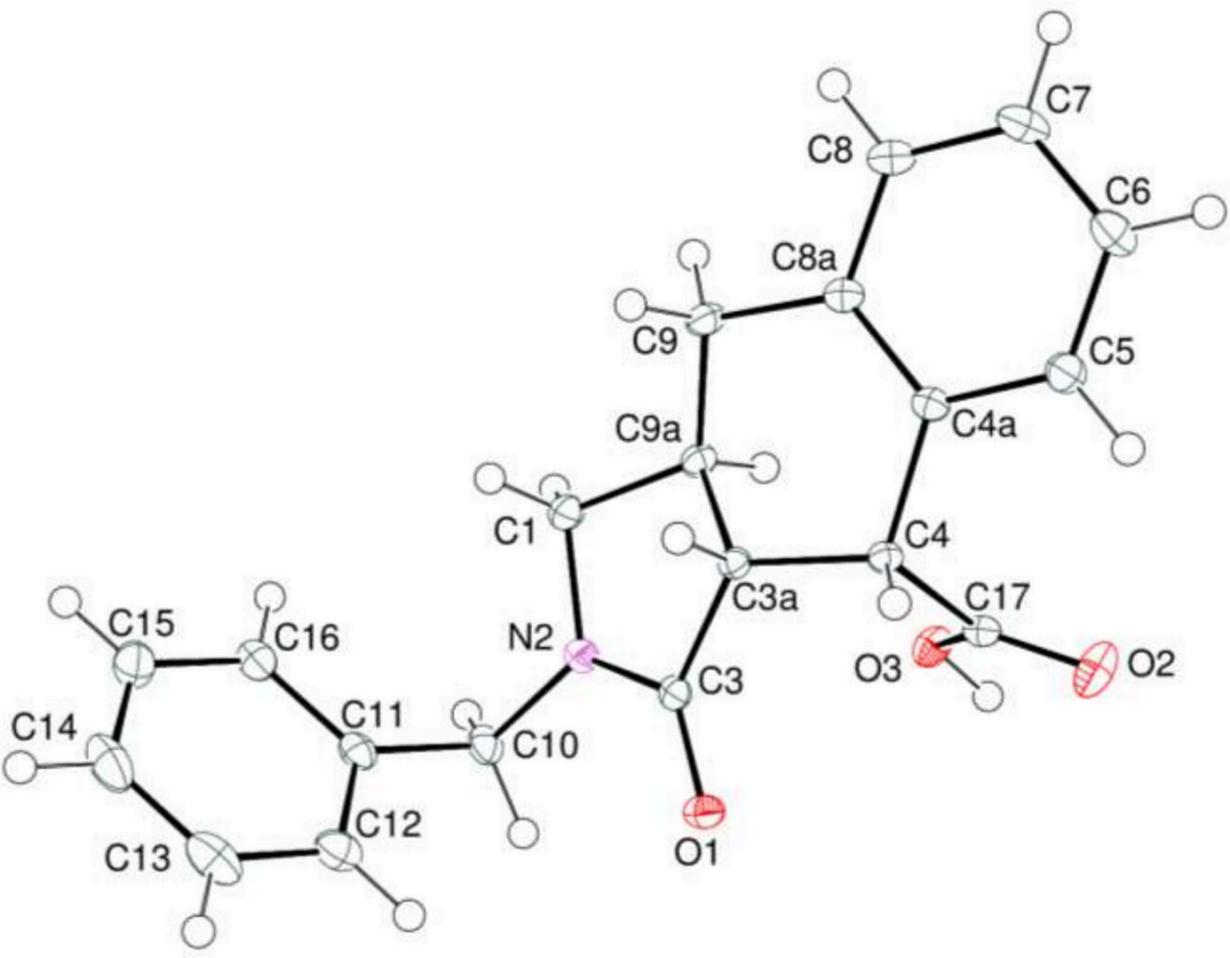
The asymmetric unit of the title compound with the atom-numbering scheme and 50% probability ellipsoids.

**Figure 3:**
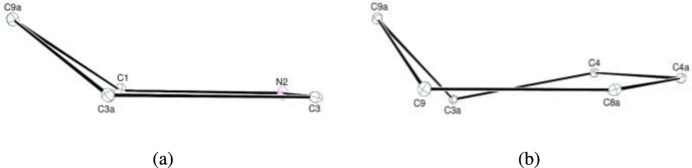
The conformations of the pyrrole (*a*) and cyclo­hexene (*b*) rings of the iso­indole ring system.

The title compound consists of a benzyl moiety bonded to the nitro­gen atom of a 1*H*-benzo[*f*]iso­indole-4-carb­oxy­lic acid group (Fig. 2 ▸). In the iso­indole group, the fused pyrrole and cyclo­hexene, *A* (N2/C1/C3/C23*A*/C9*A*) and *B* (C3*A*/C4/C4*A*/C8*A*/C9/C9*A*), rings are in envelope (Fig. 3 ▸*a*) and flattened-boat (Fig. 3 ▸*b*) conformations with puckering parameters (Cremer & Pople, 1975 ▸) φ = 252.21 (19)° (for the pyrrole ring) and *Q*_T_ = 0.5318 (13) Å, θ = 127.60 (13)° and φ = 138.60 (17)° (for the cyclo­hexene ring). Atom C9*A* is at the flap position and it is 0.5488 (12) Å away from the best least-squares plane of the other four atoms in the pyrrole ring. Atom C10 is −0.0268 (13) Å away from the best plane of the benzene, *D* (C11–C16), ring. The planar benzene, *C* (C4a/C5–C8/C8a) and *D* (C11–C16), rings are oriented at a dihedral angle of 69.64 (3)°. In the carb­oxy­lic acid moiety, the O2—C17—O3 [123.68 (11)°] bond angle is slightly widened with respect to that present in the free acid [122.2°] (Sim *et al.*, 1955 ▸), and it is reported to be 124.27 (17)° in di­aqua­bis­(2-bromo­benzoato-κ*O*)bis­(nicotinamide-κ*N*^1^)zinc(II) (Hökelek *et al.*, 2009 ▸). In a broader analysis, the observed O2—C17—O3 [123.68 (11)°] bond angle is quite normal and very similar to the median value of 124.36° calculated from *ca.* 2700 deposited structures in the CSD (Groom *et al.*, 2016 ▸).

## 3. Supra­molecular features

**Figure 4:**
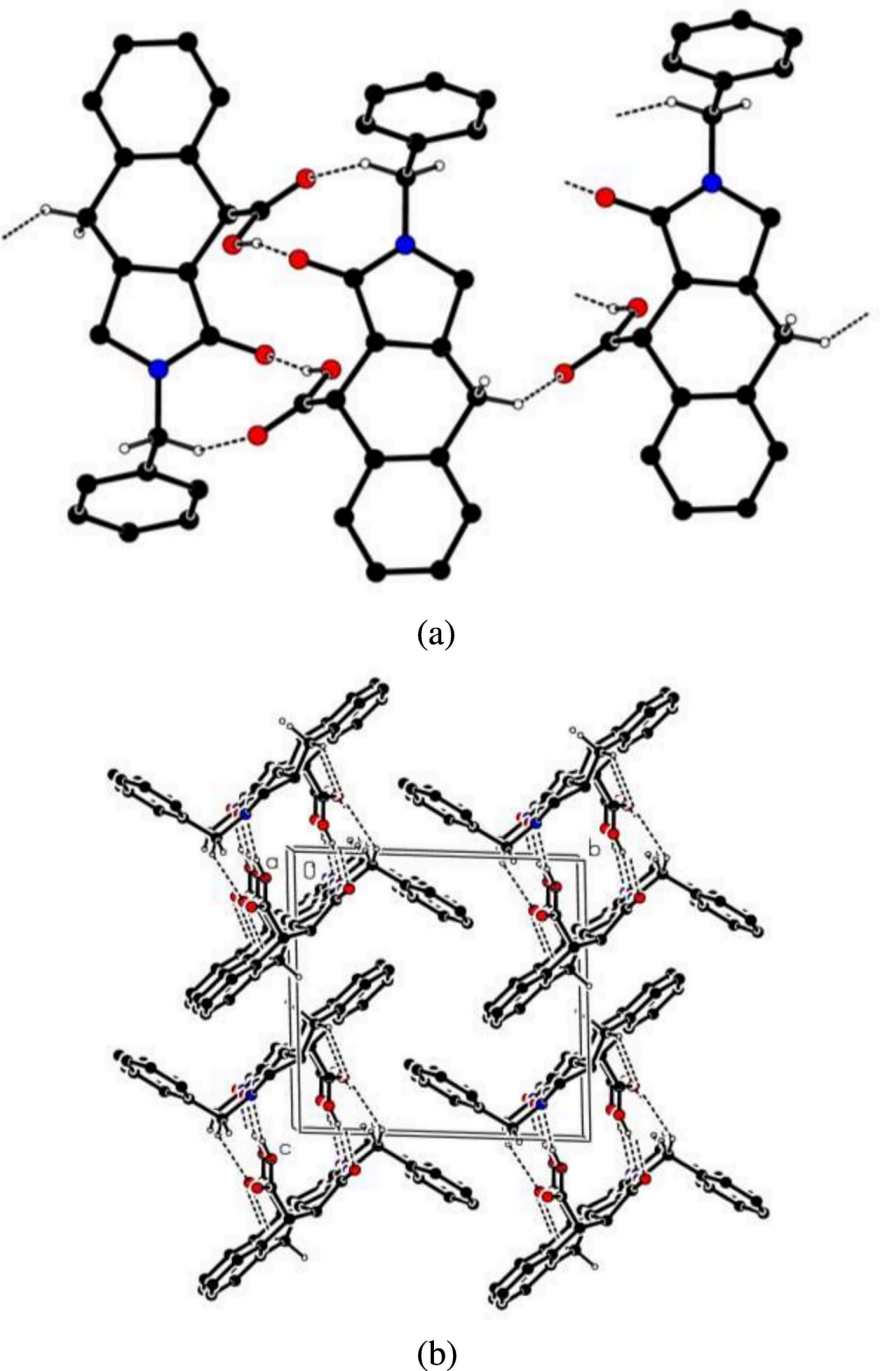
Partial packing diagrams of the title compound showing the O—H⋯O and C—H⋯O hydrogen bonds as dashed lines with (*a*) the 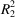
(9) and 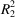
(14) ring motifs and (*b*) the infinite double-chains viewed along the *a*-axis direction.

In the crystal, O—H⋯O and C—H⋯O hydrogen bonds (Table 1 ▸) link the mol­ecules, enclosing 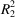
(14) and 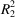
(9) ring motifs (Etter *et al.*, 1990 ▸) (Fig. 4 ▸*a*), into infinite double-chains along the *a*-axis direction (Fig. 4 ▸*b*). π–π stacking inter­actions between the *D* rings with centroid-to-centroid distance, α and slippage values of 3.8650 (13) Å, 0.00 (7)° and 1.710 Å, respectively (Table 1 ▸) may help consolidate the packing. More notably, the C—H⋯π(ring) inter­actions (Table 1 ▸) with a C—H⋯centroid distance of 2.53 Å between atom C3*A* and the *C* ring are very efficiently arranged and bidirectional between the two mol­ecules, giving rise to the formation of additional pairs to those generated by the O—H⋯O contacts. The C—H⋯π(ring) inter­actions link the infinite double chains in the *c*-axis direction resulting from hydrogen bonding, leading to broad sheets in the *ac* plane.

**Figure 5:**
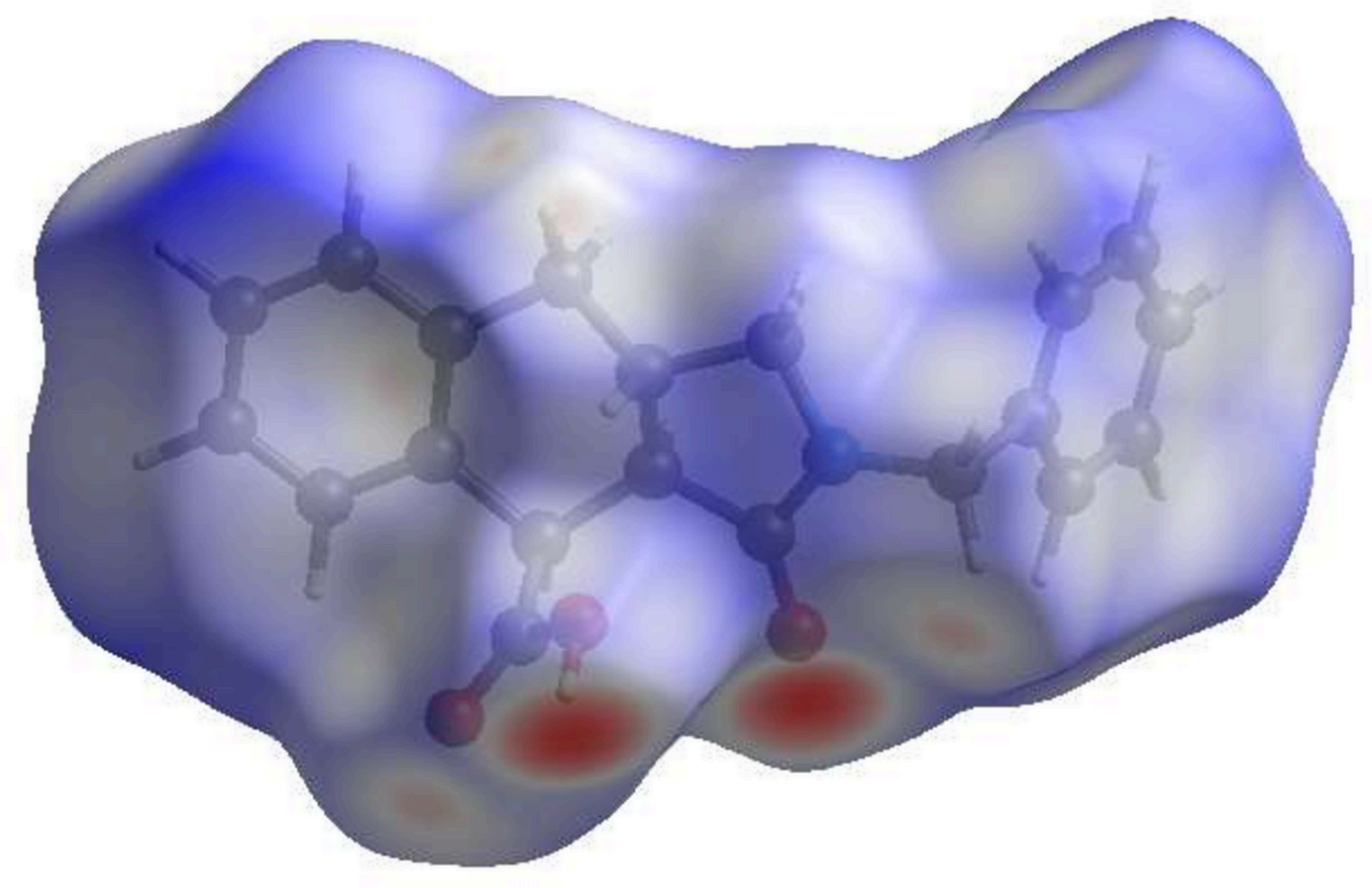
View of the three-dimensional Hirshfeld surface for the title compound plotted over *d*_norm_ in the range −0.7390 to 1.6319 a.u.

**Figure 6:**
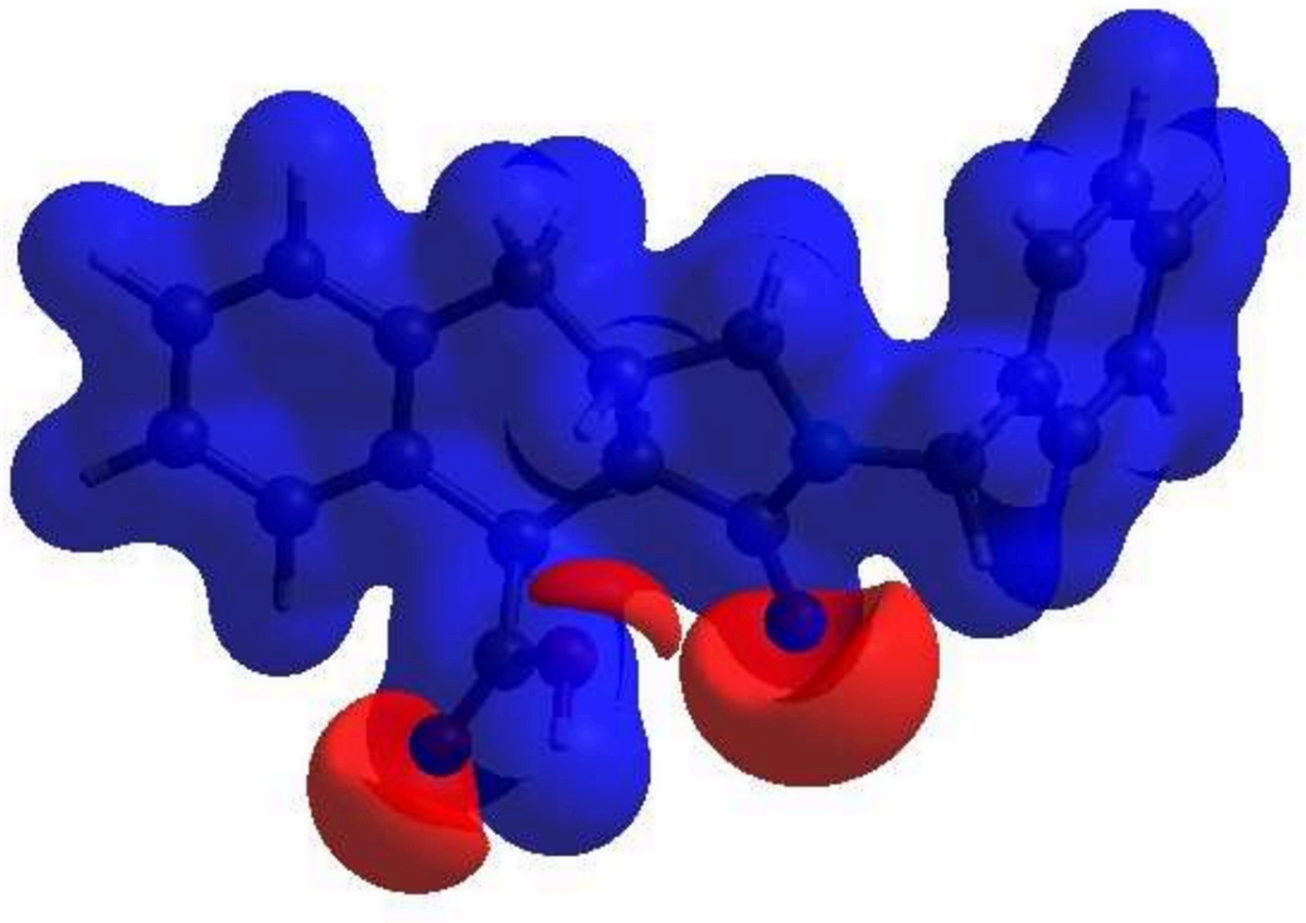
View of the three-dimensional Hirshfeld surface of the title compound plotted over the electrostatic potential in the range of −0.0500 to 0.0500 a.u. using the STO-3 G basis set at the Hartree–Fock level of theory. Hydrogen-bond donors and acceptors are shown as blue and red regions around the atoms, corresponding to positive and negative potentials, respectively.

**Figure 7:**
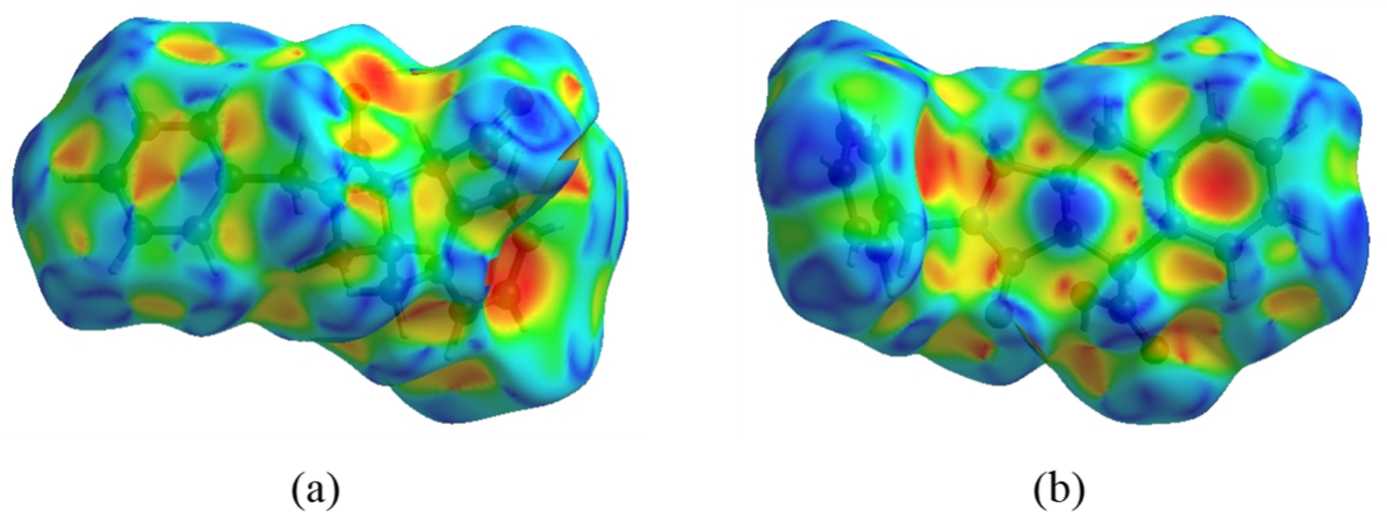
Two orientations of the shape-index surface showing (*a*) the π–π and (*b*) the C—H⋯π(ring) inter­actions.

The inter­molecular inter­actions in the crystal are visualized through a Hirshfeld surface (HS) analysis using *CrystalExplorer* 17.5 (Spackman *et al.*, 2021 ▸). Fig. 5 ▸ shows the Hirshfeld surface as impacted by several adjacent mol­ecules in the crystal. The white surface indicates contacts with distances equal to the sum of van der Waals radii, and the red and blue colours indicate distances shorter (in close contact) or longer (more distant atom) than the van der Waals radii, respectively. The red spots indicate their roles as the respective donors and/or acceptors in the hydrogen bonding patterns, as discussed above; they also appear as the blue and red regions corresponding to positive and negative potentials on the HS mapped over the electrostatic potential as shown in Fig. 6 ▸. The blue and red regions indicate positive (hydrogen-bond donor) and negative (hydrogen-bond acceptor) electrostatic potentials. The π–π stacking and C—H⋯π(ring) inter­actions (Table 1 ▸) are indicated in Fig. 7 ▸*a*,*b* by the presence of adjacent red and blue triangles and red π-holes, respectively. In Fig. 7 ▸*b*, the extensive blue dot for the C—H from the inter­action with the similarly notable red π-hole of the adjacent mol­ecule can be very clearly seen. This suggests that this contact is of significant importance for the packing.

**Figure 8:**
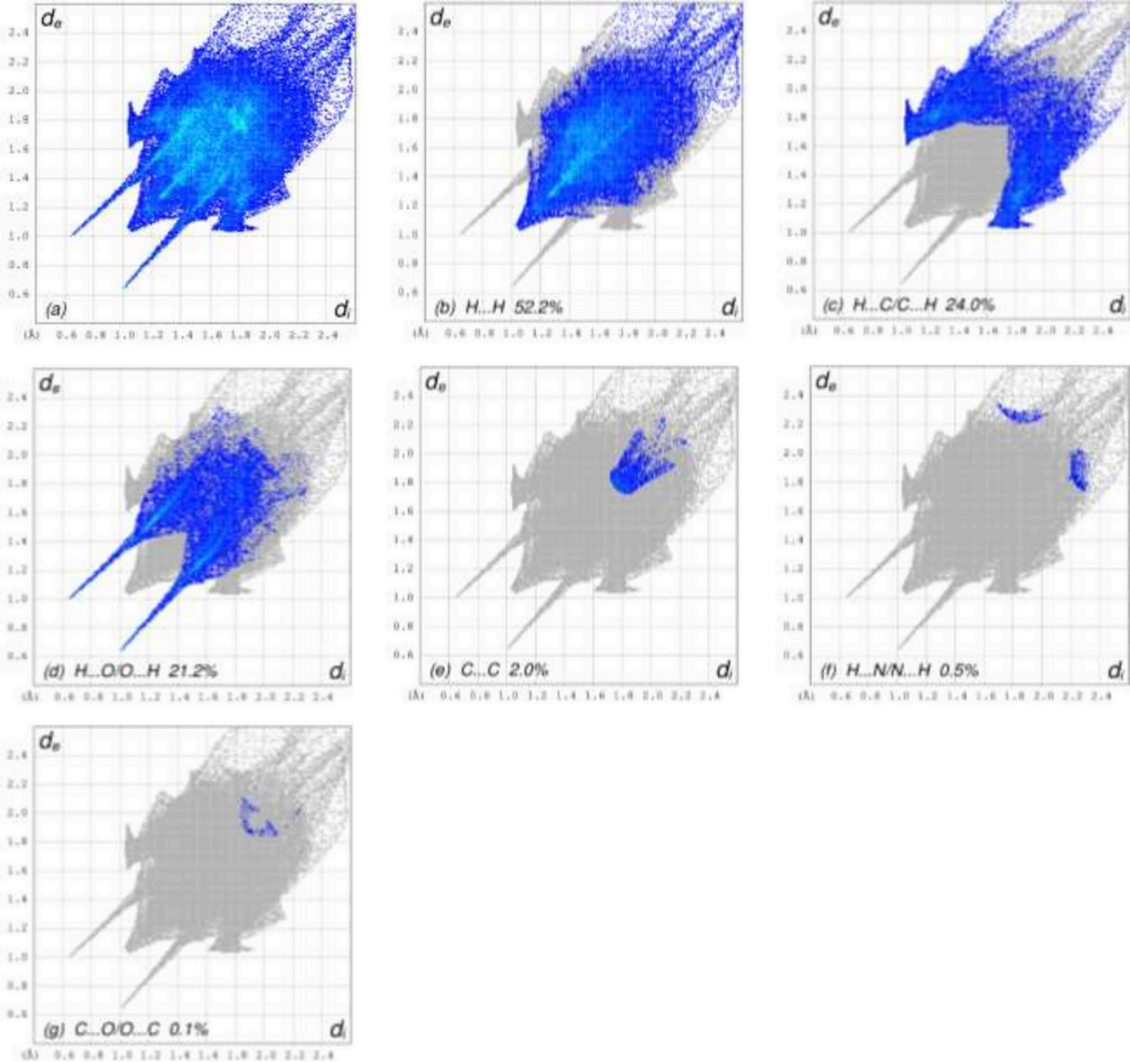
The full two-dimensional fingerprint plots for the title mol­ecule, showing (*a*) all inter­actions, and those delineated into (*b*) H⋯H, (*c*) H⋯C/C⋯H, (*d*) H⋯O/O⋯H, (*e*) C⋯C, (*f*) H⋯N/N⋯H and (*g*) C⋯O/O⋯C inter­actions. The *d*_i_ and *d*_e_ values are the closest inter­nal and external distances (in Å) from given points on the Hirshfeld surface.

The overall two-dimensional fingerprint plot is shown in Fig. 8 ▸*a* and those delineated into H⋯H, H⋯C/C⋯H, H⋯O/O⋯H, C⋯C, H⋯N/N⋯H and C⋯O/O⋯C inter­actions are illustrated in Fig. 8 ▸(*b*)–(*g*), respectively. According to the two-dimensional fingerprint plots, H⋯H, H⋯C/C⋯H and H⋯O/O⋯H contacts make the most significant contributions to the HS, at 52.2%, 24.0% and 21.2%, respectively (Fig. 8 ▸).

**Figure 9:**
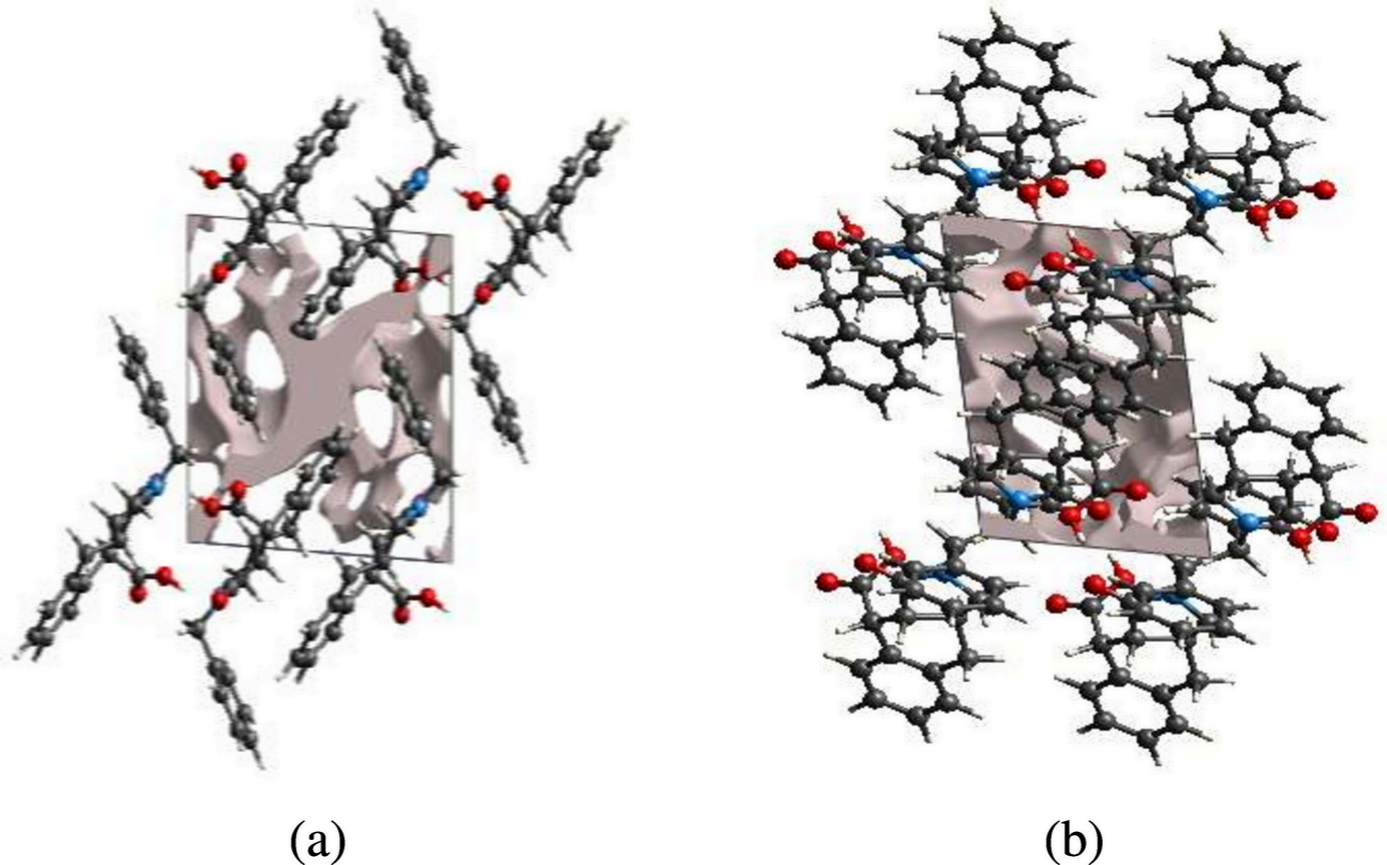
Crystal voids viewed down the crystallographic *a*-axis (*a*) and *b*-axis (*b*) directions.

The strength of the crystal depends on the tight packing of the mol­ecules and having concomittantly insignificant voids only. For checking the strength of the crystal, a void analysis was performed. The volume of the crystal voids (Fig. 9 ▸*a*,*b*) and the percentage of free space in the unit cell were calculated as 92.25 Å^3^ and 11.52%, respectively. Thus, the crystal packing appears rather compact.

The inter­molecular inter­action energies were calculated using the CE–B3LYP/6–31G(d,p) energy model available in *CrystalExplorer 17.5* (Spackman *et al.*, 2021 ▸), where a cluster of mol­ecules is generated by applying crystallographic symmetry operations with respect to a selected central mol­ecule within the radius of 3.8 Å by default. The total inter­molecular energy (*E*_tot_) is the sum of electrostatic (*E*_ele_), polarization (*E*_pol_), dispersion (*E*_dis_) and exchange-repulsion (*E*_rep_) energies (Turner *et al.*, 2015 ▸) with scale factors of 1.057, 0.740, 0.871 and 0.618, respectively (Mackenzie *et al.*, 2017 ▸). Hydrogen-bonding inter­action energies (in kJ mol^−1^) were calculated to be −141.4 (*E*_ele_), −42.8 (*E*_pol_), −32.0 (*E*_dis_), 119.5 (*E*_rep_) and −103.8 (*E*_tot_) for the O3—H3⋯O1 hydrogen-bonding inter­action, −30.4 (*E*_ele_), −7.5 (*E*_pol_), −96.2 (*E*_dis_), 49.8 (*E*_rep_) and −82.2 (*E*_tot_) for the C10—H10*A*⋯O2 hydrogen-bonding inter­action and −12.5 (*E*_ele_), −7.0 (*E*_pol_), −38.7 (*E*_dis_), 20.5 (*E*_rep_) and −35.5 (*E*_tot_) for the C9—H9*B⋯*O2 hydrogen-bonding inter­action.

**Figure 10:**
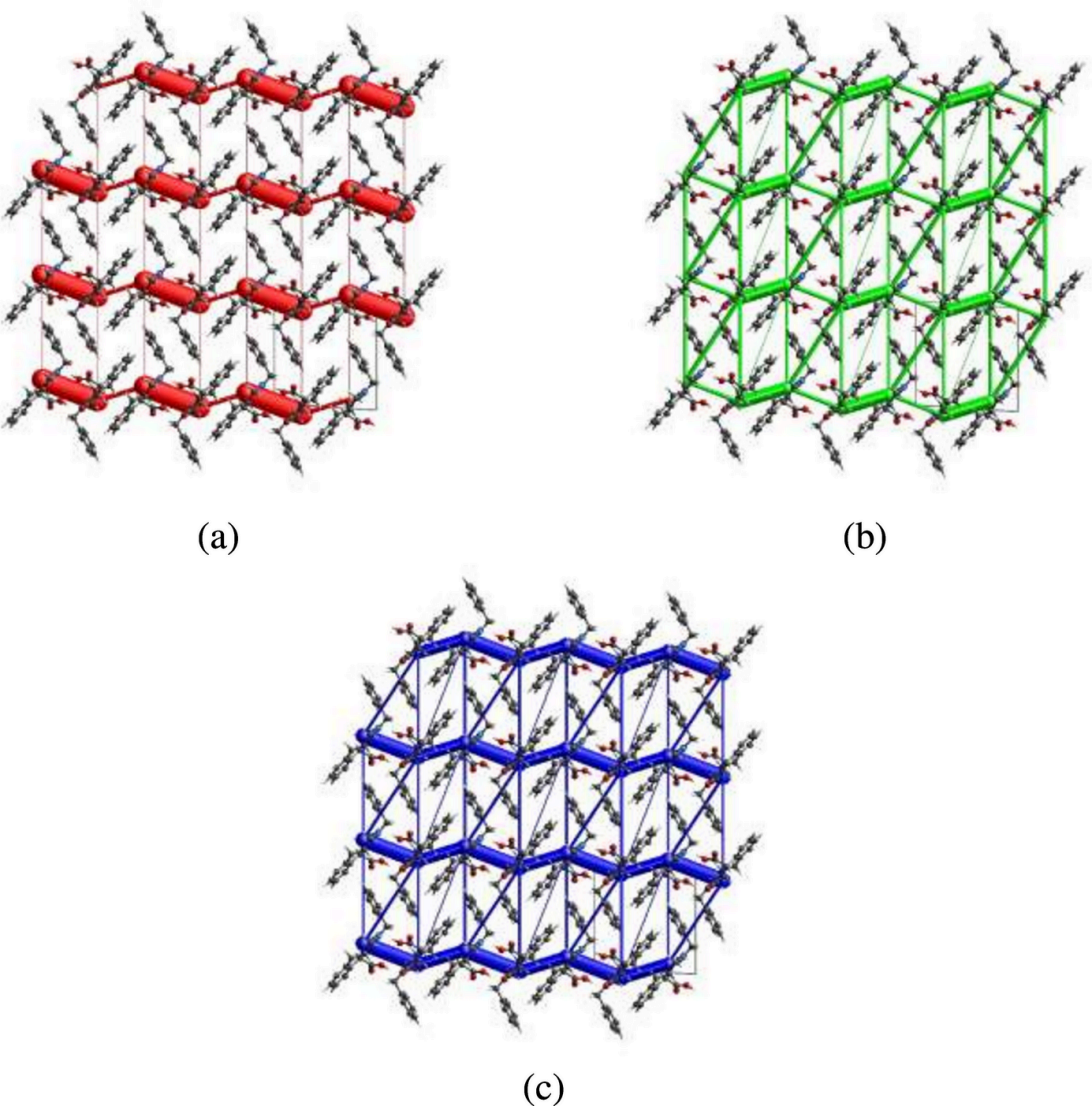
The energy frameworks for a cluster of mol­ecules of the title compound viewed down the *a*-axis showing (*a*) the electrostatic energy, (*b*) the dispersion energy and (*c*) the total energy diagrams. The cylindrical radius is proportional to the relative strength of the corresponding energies and they were adjusted to the same scale factor of 80 with cut-off value of 5 kJ mol^−1^ within 3 × 3 × 3 unit cells.

Energy frameworks combine the calculation of inter­molecular inter­action energies with a graphical representation of their magnitudes, in which they were constructed for *E*_ele_ (red cylinders), *E*_dis_ (green cylinders) and *E*_tot_ (blue cylinders) (Fig. 10 ▸*a*,*b*,*c*). The evaluations of the electrostatic, dispersion and total energy frameworks indicate that the electrostatic energy contributions dominate in the crystal structure of the title compound.

## 4. Synthesis and crystallization

(2*E*)-*N*-Benzyl-3-phenyl­prop-2-en-1-amine (**2**) (0.67 g, 3.00 mmol) was dissolved in aceto­nitrile (15 ml), and maleic anhydride (0.29 g, 3.00 mmol) was added. The reaction mixture was refluxed for 8 h. Upon cooling to room temperature, the resulting solid was collected by filtration, washed with diethyl ether (2 × 10 ml), and air-dried to afford the target compound (**1**) as white crystalline powder (0.47 g, 1.47 mmol, 49%, m.p. 503-505 K). A single crystal suitable for X-ray diffraction analysis was found in the obtained crystalline material. ^1^H NMR (700 MHz, DMSO-*d*_6_, 298 K) (*J*, Hz): δ 12.49 (*br. s*, 1H, COOH), 7.47–7.46 (*m*, 1H, H_arom_), 7.35–7.33 (*m*, 2H, H_arom_), 7.28–7.26 (*m*, 3H, H_arom_), 7.20–7.16 (*m*, 3H, H_arom_), 4.50 (*d*, *J* = 15.3, 1H, H10*A*—NCH_2_Ph), 4.36 (*d*, *J* = 15.3, 1H, H10*B*—NCH_2_Ph), 4.00 (*dd*, *J* = 6.1, 1H, H4_methine_), 3.42–3.39 (*m*, 1H, H1*A*_methyl­ene_), 3.09–3.07 (*m*, 1H, H1*B*_methyl­ene_), 3.05–3.00 (*m*, 1H, H9*C*_methine_), 2.93 (*dd*, *J* = 4.3, 15.7, 1H, H9*A*_methyl­ene_), 2.70 (*dd*, *J* = 12.4, 15.0, 1H, H9*B*_methyl­ene_), 2.40 (*dd*, *J* = 5.5, 12.6, 1H, H3*A*_methine_) ppm. ^13^C{^1^H} NMR (176 MHz, DMSO-*d*_6_, 298 K): δ 173.15, 173.12, 137.3, 136.7, 133.0, 130.1, 128.5 (2C), 127.4 (2C), 127.1, 127.0, 126.1, 50.2, 45.7, 45.3, 42.4, 32.7, 32.2 ppm. IR (KBr), ν (cm^−1^) 2943, 2542, 1732, 1633, 1485, 1440, 1319, 1274, 1234, 1202, 1171. Analysis calculated for C_20_H_19_NO_3_: C, 74.75; H, 5.96; N, 4.36. Found: C, 74.68; H, 6.12; N, 4.21.

## 5. Refinement

Crystal data, data collection and structure refinement details are summarized in Table 2 ▸. The OH hydrogen atom was located in a difference-Fourier map and refined isotropically. The C-bound hydrogen-atom positions were calculated geometrically at distances of 1.00 Å (for methine CH), 0.95 Å (for aromatic CH) and 0.99 Å (for methyl­ene CH) and refined using a riding model with *U*_iso_(H) = 1.2*U*_eq_(C).

## Supplementary Material

Crystal structure: contains datablock(s) I, global. DOI: 10.1107/S2056989026005189/yz2079sup1.cif

Structure factors: contains datablock(s) I. DOI: 10.1107/S2056989026005189/yz2079Isup2.hkl

Supporting information file. DOI: 10.1107/S2056989026005189/yz2079Isup3.cml

CCDC reference: 2486562

Additional supporting information:  crystallographic information; 3D view; checkCIF report

## Figures and Tables

**Table 1:** Hydrogen-bond geometry (Å, °) *Cg*3 is the centroid of the C4*A*/C5–C8/C8*A* ring.

*D*—H⋯*A*	*D*—H	H⋯*A*	*D*⋯*A*	*D*—H⋯*A*
O3—H3⋯O1^i^	0.888 (19)	1.752 (19)	2.6227 (14)	166.1 (17)
C9—H9*B*⋯O2^ii^	0.99	2.59	3.3397 (19)	133
C10—H10*A*⋯O2^i^	0.99	2.47	3.2689 (19)	138
C3*A*—H3*A*⋯*Cg*3^iii^	1.00	2.53	3.5088 (16)	166

Symmetry codes: (i) 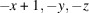; (ii) 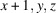; (iii) 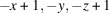.

**Table 2:** Experimental details

Crystal data	
Chemical formula	C_20_H_19_NO_3_
*M*_r_	321.36
Crystal system, space group	Triclinic, *P* 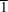
Temperature (K)	100
*a*, *b*, *c* (Å)	6.7225 (15), 11.102 (3), 11.108 (3)
α, β, γ (°)	84.894 (4), 76.890 (4), 83.580 (3)
*V* (Å^3^)	800.6 (4)
*Z*	2
Radiation type	Mo *K*α
μ (mm^−1^)	0.09
Crystal size (mm)	0.30 × 0.25 × 0.20

Data collection	
Diffractometer	Bruker APEXII area detector
Absorption correction	Multi-scan (*SADABS*; Krause *et al.*, 2015 ▸)
*T*_min_, *T*_max_	0.963, 0.980
No. of measured, independent and observed [*I* > 2σ(*I*)] reflections	13680, 5546, 4248
*R*_int_	0.022
(sin θ/λ)_max_ (Å^−1^)	0.761

Refinement	
*R*[*F*^2^ > 2σ(*F*^2^)], *wR*(*F*^2^), *S*	0.054, 0.136, 1.03
No. of reflections	5546
No. of parameters	220
H-atom treatment	H atoms treated by a mixture of independent and constrained refinement
Δρ_max_, Δρ_min_ (e Å^−3^)	0.56, −0.25

Computer programs: *APEX2* (Bruker, 2013 ▸), *SAINT* (Bruker, 2018 ▸), *SHELXT* (Sheldrick, 2015*a* ▸), *SHELXL* (Sheldrick, 2015*b* ▸) and *SHELXTL* (Sheldrick, 2008 ▸).

## supplementary crystallographic information

### (3aRS,4RS,9aRS)-2-Benzyl-3-oxo-2,3,3a,4,9,9a-hexahydro-1H-benzo[f]isoindole-4-carboxylic acid Crystal data

**Table:**

C_20_H_19_NO_3_	*Z* = 2
*M**_r_* = 321.36	*F*(000) = 340
Triclinic, *P*1	*D*_x_ = 1.333 Mg m^−^^3^
*a* = 6.7225 (15) Å	Mo *K*α radiation, λ = 0.71073 Å
*b* = 11.102 (3) Å	Cell parameters from 6018 reflections
*c* = 11.108 (3) Å	θ = 2.6–32.6°
α = 84.894 (4)°	µ = 0.09 mm^−^^1^
β = 76.890 (4)°	*T* = 100 K
γ = 83.580 (3)°	Prism, colourless
*V* = 800.6 (4) Å^3^	0.30 × 0.25 × 0.20 mm

### (3aRS,4RS,9aRS)-2-Benzyl-3-oxo-2,3,3a,4,9,9a-hexahydro-1H-benzo[f]isoindole-4-carboxylic acid Data collection

**Table:**

Bruker APEXII area detector diffractometer	4248 reflections with *I* > 2σ(*I*)
Radiation source: fine-focus sealed tube	*R*_int_ = 0.022
φ and ω scans	θ_max_ = 32.8°, θ_min_ = 1.9°
Absorption correction: multi-scan (SADABS; Krause *et al.*, 2015)	*h* = −10→9
*T*_min_ = 0.963, *T*_max_ = 0.980	*k* = −16→16
13680 measured reflections	*l* = −16→16
5546 independent reflections	

### (3aRS,4RS,9aRS)-2-Benzyl-3-oxo-2,3,3a,4,9,9a-hexahydro-1H-benzo[f]isoindole-4-carboxylic acid Refinement

**Table:**

Refinement on *F*^2^	Primary atom site location: difference Fourier map
Least-squares matrix: full	Secondary atom site location: difference Fourier map
*R*[*F*^2^ > 2σ(*F*^2^)] = 0.054	Hydrogen site location: mixed
*wR*(*F*^2^) = 0.136	H atoms treated by a mixture of independent and constrained refinement
*S* = 1.03	*w* = 1/[σ^2^(*F*_o_^2^) + (0.0621*P*)^2^ + 0.37*P*] where *P* = (*F*_o_^2^ + 2*F*_c_^2^)/3
5546 reflections	(Δ/σ)_max_ < 0.001
220 parameters	Δρ_max_ = 0.56 e Å^−^^3^
0 restraints	Δρ_min_ = −0.25 e Å^−^^3^

### (3aRS,4RS,9aRS)-2-Benzyl-3-oxo-2,3,3a,4,9,9a-hexahydro-1H-benzo[f]isoindole-4-carboxylic acid Special details

**Table:**

Geometry. All esds (except the esd in the dihedral angle between two l.s. planes) are estimated using the full covariance matrix. The cell esds are taken into account individually in the estimation of esds in distances, angles and torsion angles; correlations between esds in cell parameters are only used when they are defined by crystal symmetry. An approximate (isotropic) treatment of cell esds is used for estimating esds involving l.s. planes.

### (3aRS,4RS,9aRS)-2-Benzyl-3-oxo-2,3,3a,4,9,9a-hexahydro-1H-benzo[f]isoindole-4-carboxylic acid Fractional atomic coordinates and isotropic or equivalent isotropic displacement parameters (Å^2^)

**Table:**

	*x*	*y*	*z*	*U*_iso_*/*U*_eq_	
O1	0.47170 (13)	0.17245 (8)	0.11760 (8)	0.01417 (18)	
O2	0.29170 (15)	−0.16976 (10)	0.18918 (9)	0.0225 (2)	
O3	0.59880 (14)	−0.11167 (9)	0.09065 (8)	0.01539 (19)	
H3	0.565 (3)	−0.1419 (16)	0.0275 (17)	0.023*	
C1	0.95288 (19)	0.10015 (12)	0.20139 (12)	0.0154 (2)	
H1A	0.968369	0.153351	0.264981	0.019*	
H1B	1.089892	0.075125	0.150450	0.019*	
N2	0.81469 (16)	0.16053 (10)	0.12427 (10)	0.0138 (2)	
C3	0.62090 (18)	0.13013 (11)	0.16167 (11)	0.0113 (2)	
C3A	0.61601 (17)	0.04056 (11)	0.27320 (11)	0.0104 (2)	
H3A	0.582024	0.090138	0.347302	0.012*	
C4	0.46745 (17)	−0.05682 (11)	0.30006 (10)	0.0105 (2)	
H4	0.329661	−0.016392	0.337862	0.013*	
C4A	0.52742 (18)	−0.15235 (11)	0.39842 (11)	0.0115 (2)	
C5	0.39286 (19)	−0.24037 (12)	0.45008 (12)	0.0154 (2)	
H5	0.269579	−0.240813	0.422016	0.018*	
C6	0.4354 (2)	−0.32699 (13)	0.54135 (13)	0.0197 (3)	
H6	0.341939	−0.385686	0.575476	0.024*	
C7	0.6163 (2)	−0.32686 (13)	0.58225 (13)	0.0204 (3)	
H7	0.646653	−0.385144	0.645110	0.024*	
C8	0.7520 (2)	−0.24141 (12)	0.53094 (12)	0.0169 (2)	
H8	0.875866	−0.242600	0.558770	0.020*	
C8A	0.71126 (18)	−0.15299 (11)	0.43877 (11)	0.0124 (2)	
C9	0.86880 (18)	−0.06341 (12)	0.38610 (11)	0.0140 (2)	
H9A	0.853288	0.002468	0.442969	0.017*	
H9B	1.008710	−0.105233	0.378223	0.017*	
C9A	0.83988 (18)	−0.01002 (11)	0.26026 (11)	0.0121 (2)	
H9C	0.871886	−0.075616	0.200736	0.015*	
C10	0.8754 (2)	0.26392 (12)	0.03716 (12)	0.0156 (2)	
H10A	0.784425	0.277362	−0.022431	0.019*	
H10B	1.017686	0.245513	−0.010136	0.019*	
C11	0.86246 (19)	0.37789 (11)	0.10473 (11)	0.0144 (2)	
C12	0.6812 (2)	0.45538 (13)	0.12481 (13)	0.0196 (3)	
H12	0.567626	0.436842	0.094474	0.023*	
C13	0.6653 (2)	0.55930 (13)	0.18871 (14)	0.0255 (3)	
H13	0.541318	0.611417	0.201913	0.031*	
C14	0.8304 (3)	0.58691 (13)	0.23320 (14)	0.0268 (3)	
H14	0.819651	0.657971	0.276873	0.032*	
C15	1.0114 (2)	0.51058 (13)	0.21384 (14)	0.0242 (3)	
H15	1.124517	0.529574	0.244299	0.029*	
C16	1.0277 (2)	0.40614 (12)	0.14984 (13)	0.0182 (3)	
H16	1.151744	0.354112	0.136972	0.022*	
C17	0.44251 (18)	−0.11850 (11)	0.18782 (11)	0.0124 (2)	

### (3aRS,4RS,9aRS)-2-Benzyl-3-oxo-2,3,3a,4,9,9a-hexahydro-1H-benzo[f]isoindole-4-carboxylic acid Atomic displacement parameters (Å^2^)

**Table:**

	*U*^11^	*U*^22^	*U*^33^	*U*^12^	*U*^13^	*U*^23^
O1	0.0157 (4)	0.0146 (4)	0.0127 (4)	0.0013 (3)	−0.0054 (3)	−0.0011 (3)
O2	0.0173 (4)	0.0333 (6)	0.0190 (5)	−0.0097 (4)	−0.0027 (4)	−0.0077 (4)
O3	0.0151 (4)	0.0210 (5)	0.0107 (4)	−0.0035 (3)	−0.0021 (3)	−0.0039 (3)
C1	0.0129 (5)	0.0164 (6)	0.0179 (6)	−0.0040 (4)	−0.0055 (4)	0.0026 (5)
N2	0.0141 (5)	0.0136 (5)	0.0140 (5)	−0.0026 (4)	−0.0040 (4)	0.0019 (4)
C3	0.0145 (5)	0.0103 (5)	0.0093 (5)	−0.0009 (4)	−0.0028 (4)	−0.0022 (4)
C3A	0.0109 (5)	0.0111 (5)	0.0096 (5)	−0.0018 (4)	−0.0029 (4)	−0.0005 (4)
C4	0.0102 (5)	0.0116 (5)	0.0096 (5)	−0.0007 (4)	−0.0021 (4)	−0.0002 (4)
C4A	0.0127 (5)	0.0117 (5)	0.0098 (5)	0.0001 (4)	−0.0020 (4)	−0.0007 (4)
C5	0.0146 (5)	0.0152 (6)	0.0160 (6)	−0.0016 (4)	−0.0030 (4)	0.0005 (4)
C6	0.0201 (6)	0.0159 (6)	0.0206 (6)	−0.0019 (5)	−0.0013 (5)	0.0048 (5)
C7	0.0223 (6)	0.0185 (6)	0.0182 (6)	0.0013 (5)	−0.0044 (5)	0.0066 (5)
C8	0.0169 (6)	0.0194 (6)	0.0144 (6)	0.0018 (5)	−0.0057 (4)	0.0009 (5)
C8A	0.0124 (5)	0.0140 (5)	0.0102 (5)	0.0009 (4)	−0.0021 (4)	−0.0011 (4)
C9	0.0120 (5)	0.0173 (6)	0.0135 (5)	−0.0015 (4)	−0.0052 (4)	0.0005 (4)
C9A	0.0103 (5)	0.0142 (5)	0.0119 (5)	−0.0009 (4)	−0.0030 (4)	−0.0002 (4)
C10	0.0198 (6)	0.0136 (6)	0.0130 (5)	−0.0051 (5)	−0.0019 (4)	0.0016 (4)
C11	0.0174 (6)	0.0128 (5)	0.0120 (5)	−0.0028 (4)	−0.0014 (4)	0.0020 (4)
C12	0.0184 (6)	0.0178 (6)	0.0206 (6)	−0.0006 (5)	−0.0029 (5)	0.0039 (5)
C13	0.0280 (7)	0.0167 (6)	0.0262 (7)	0.0035 (5)	0.0021 (6)	0.0017 (5)
C14	0.0428 (9)	0.0139 (6)	0.0215 (7)	−0.0022 (6)	−0.0016 (6)	−0.0027 (5)
C15	0.0342 (8)	0.0191 (7)	0.0226 (7)	−0.0066 (6)	−0.0113 (6)	−0.0005 (5)
C16	0.0200 (6)	0.0144 (6)	0.0206 (6)	−0.0021 (5)	−0.0056 (5)	0.0004 (5)
C17	0.0132 (5)	0.0127 (5)	0.0114 (5)	0.0006 (4)	−0.0037 (4)	−0.0006 (4)

### (3aRS,4RS,9aRS)-2-Benzyl-3-oxo-2,3,3a,4,9,9a-hexahydro-1H-benzo[f]isoindole-4-carboxylic acid Geometric parameters (Å, º)

**Table:**

O1—C3	1.2421 (14)	C7—C8	1.385 (2)
O2—C17	1.2134 (15)	C7—H7	0.9500
O3—C17	1.3280 (15)	C8—C8A	1.4054 (17)
O3—H3	0.888 (19)	C8—H8	0.9500
C1—N2	1.4717 (16)	C8A—C9	1.5179 (18)
C1—C9A	1.5300 (18)	C9—C9A	1.5143 (17)
C1—H1A	0.9900	C9—H9A	0.9900
C1—H1B	0.9900	C9—H9B	0.9900
N2—C3	1.3459 (16)	C9A—H9C	1.0000
N2—C10	1.4657 (16)	C10—C11	1.5122 (18)
C3—C3A	1.5147 (17)	C10—H10A	0.9900
C3A—C4	1.5179 (17)	C10—H10B	0.9900
C3A—C9A	1.5258 (16)	C11—C16	1.3934 (19)
C3A—H3A	1.0000	C11—C12	1.3971 (18)
C4—C17	1.5244 (17)	C12—C13	1.389 (2)
C4—C4A	1.5423 (17)	C12—H12	0.9500
C4—H4	1.0000	C13—C14	1.386 (2)
C4A—C5	1.4018 (17)	C13—H13	0.9500
C4A—C8A	1.4061 (17)	C14—C15	1.389 (2)
C5—C6	1.3896 (18)	C14—H14	0.9500
C5—H5	0.9500	C15—C16	1.395 (2)
C6—C7	1.392 (2)	C15—H15	0.9500
C6—H6	0.9500	C16—H16	0.9500
			
C17—O3—H3	108.1 (11)	C8—C8A—C9	118.55 (11)
N2—C1—C9A	101.85 (9)	C4A—C8A—C9	122.92 (11)
N2—C1—H1A	111.4	C9A—C9—C8A	109.98 (10)
C9A—C1—H1A	111.4	C9A—C9—H9A	109.7
N2—C1—H1B	111.4	C8A—C9—H9A	109.7
C9A—C1—H1B	111.4	C9A—C9—H9B	109.7
H1A—C1—H1B	109.3	C8A—C9—H9B	109.7
C3—N2—C10	124.17 (11)	H9A—C9—H9B	108.2
C3—N2—C1	113.24 (10)	C9—C9A—C3A	108.19 (9)
C10—N2—C1	120.97 (10)	C9—C9A—C1	118.81 (10)
O1—C3—N2	126.58 (11)	C3A—C9A—C1	101.80 (10)
O1—C3—C3A	126.31 (11)	C9—C9A—H9C	109.2
N2—C3—C3A	107.00 (10)	C3A—C9A—H9C	109.2
C3—C3A—C4	120.42 (10)	C1—C9A—H9C	109.2
C3—C3A—C9A	103.52 (9)	N2—C10—C11	110.98 (10)
C4—C3A—C9A	113.53 (10)	N2—C10—H10A	109.4
C3—C3A—H3A	106.1	C11—C10—H10A	109.4
C4—C3A—H3A	106.1	N2—C10—H10B	109.4
C9A—C3A—H3A	106.1	C11—C10—H10B	109.4
C3A—C4—C17	115.75 (10)	H10A—C10—H10B	108.0
C3A—C4—C4A	109.62 (9)	C16—C11—C12	119.07 (12)
C17—C4—C4A	110.39 (10)	C16—C11—C10	121.05 (11)
C3A—C4—H4	106.9	C12—C11—C10	119.86 (12)
C17—C4—H4	106.9	C13—C12—C11	120.64 (13)
C4A—C4—H4	106.9	C13—C12—H12	119.7
C5—C4A—C8A	119.09 (11)	C11—C12—H12	119.7
C5—C4A—C4	118.79 (10)	C14—C13—C12	119.98 (13)
C8A—C4A—C4	122.12 (10)	C14—C13—H13	120.0
C6—C5—C4A	121.61 (12)	C12—C13—H13	120.0
C6—C5—H5	119.2	C13—C14—C15	119.92 (14)
C4A—C5—H5	119.2	C13—C14—H14	120.0
C5—C6—C7	119.30 (12)	C15—C14—H14	120.0
C5—C6—H6	120.3	C14—C15—C16	120.22 (14)
C7—C6—H6	120.3	C14—C15—H15	119.9
C8—C7—C6	119.74 (12)	C16—C15—H15	119.9
C8—C7—H7	120.1	C11—C16—C15	120.17 (13)
C6—C7—H7	120.1	C11—C16—H16	119.9
C7—C8—C8A	121.72 (12)	C15—C16—H16	119.9
C7—C8—H8	119.1	O2—C17—O3	123.68 (11)
C8A—C8—H8	119.1	O2—C17—C4	121.90 (11)
C8—C8A—C4A	118.53 (11)	O3—C17—C4	114.43 (10)
			
C9A—C1—N2—C3	21.91 (13)	C4—C4A—C8A—C9	−2.30 (18)
C9A—C1—N2—C10	−172.05 (10)	C8—C8A—C9—C9A	158.54 (11)
C10—N2—C3—O1	10.3 (2)	C4A—C8A—C9—C9A	−20.69 (16)
C1—N2—C3—O1	175.87 (12)	C8A—C9—C9A—C3A	52.82 (13)
C10—N2—C3—C3A	−166.01 (11)	C8A—C9—C9A—C1	168.06 (10)
C1—N2—C3—C3A	−0.49 (14)	C3—C3A—C9A—C9	159.39 (10)
O1—C3—C3A—C4	34.16 (17)	C4—C3A—C9A—C9	−68.34 (13)
N2—C3—C3A—C4	−149.48 (11)	C3—C3A—C9A—C1	33.45 (11)
O1—C3—C3A—C9A	162.28 (12)	C4—C3A—C9A—C1	165.73 (10)
N2—C3—C3A—C9A	−21.35 (12)	N2—C1—C9A—C9	−151.69 (11)
C3—C3A—C4—C17	41.27 (15)	N2—C1—C9A—C3A	−33.08 (11)
C9A—C3A—C4—C17	−82.19 (12)	C3—N2—C10—C11	89.25 (14)
C3—C3A—C4—C4A	166.90 (10)	C1—N2—C10—C11	−75.21 (14)
C9A—C3A—C4—C4A	43.44 (13)	N2—C10—C11—C16	87.71 (14)
C3A—C4—C4A—C5	170.64 (10)	N2—C10—C11—C12	−91.00 (14)
C17—C4—C4A—C5	−60.71 (14)	C16—C11—C12—C13	0.07 (19)
C3A—C4—C4A—C8A	−8.61 (15)	C10—C11—C12—C13	178.80 (12)
C17—C4—C4A—C8A	120.04 (12)	C11—C12—C13—C14	0.0 (2)
C8A—C4A—C5—C6	0.94 (18)	C12—C13—C14—C15	0.0 (2)
C4—C4A—C5—C6	−178.33 (11)	C13—C14—C15—C16	0.0 (2)
C4A—C5—C6—C7	−0.3 (2)	C12—C11—C16—C15	−0.13 (19)
C5—C6—C7—C8	−0.6 (2)	C10—C11—C16—C15	−178.86 (12)
C6—C7—C8—C8A	0.7 (2)	C14—C15—C16—C11	0.1 (2)
C7—C8—C8A—C4A	−0.04 (19)	C3A—C4—C17—O2	−158.11 (12)
C7—C8—C8A—C9	−179.30 (12)	C4A—C4—C17—O2	76.65 (14)
C5—C4A—C8A—C8	−0.77 (17)	C3A—C4—C17—O3	22.31 (15)
C4—C4A—C8A—C8	178.47 (11)	C4A—C4—C17—O3	−102.94 (12)
C5—C4A—C8A—C9	178.45 (11)		

### (3aRS,4RS,9aRS)-2-Benzyl-3-oxo-2,3,3a,4,9,9a-hexahydro-1H-benzo[f]isoindole-4-carboxylic acid Hydrogen-bond geometry (Å, º)

*Cg*3 is the centroid of the C4*A*/C5–C8/C8*A* ring.

**Table:**

*D*—H···*A*	*D*—H	H···*A*	*D*···*A*	*D*—H···*A*
O3—H3···O1^i^	0.888 (19)	1.752 (19)	2.6227 (14)	166.1 (17)
C9—H9*B*···O2^ii^	0.99	2.59	3.3397 (19)	133
C10—H10*A*···O2^i^	0.99	2.47	3.2689 (19)	138
C3*A*—H3*A*···*Cg*3^iii^	1.00	2.53	3.5088 (16)	166

Symmetry codes: (i) −*x*+1, −*y*, −*z*; (ii) *x*+1, *y*, *z*; (iii) −*x*+1, −*y*, −*z*+1.
